# Relationship between Kellgren-Lawrence score and 3D kinematic gait analysis of patients with medial knee osteoarthritis using a new gait system

**DOI:** 10.1038/s41598-017-04390-5

**Published:** 2017-06-22

**Authors:** Xiaolong Zeng, Limin Ma, Zefeng Lin, Wenhan Huang, Zhiqiang Huang, Yu Zhang, Chuanbin Mao

**Affiliations:** 1 Guangzhou Medical University, Xinzao, Panyu District, Guangzhou, 511436 Guangdong China; 2 Department of Orthopedics, Guangdong Key Lab of Orthopedic Technology and Implant, Guangzhou General Hospital of Guangzhou Military Command, 111 Liuhua Road, Guangzhou, 510010 Guangdong China; 3 Department of Orthopaedics and Traumatology, Prince of Wales Hospital, The Chinese University of Hong Kong, Prince of Wales Hospital, 30-32 Ngan Shing Street, Shatin, New Territories, Hong Kong China; 4 School of Materials Science and Engineering, Zhejiang University, Hangzhou, Zhejiang 310027 China; 5 Department of Chemistry and Biochemistry, Stephenson Life Sciences Research Center, University of Oklahoma, Norman, OK 73019 USA

## Abstract

Knee osteoarthritis (KOA) is reported to have characteristic kinematics during walking. However, the relationship between Kellgren-Lawrence (K/L) score and the 3D kinematic gait of patients with medial KOA remains unclear. Here, ninety-seven patients with medial KOA and thirty-eight asymptomatic participants were involved. Patients with medial KOA were divided into early, moderate, and severe KOA based on the K/L score. Through kinematic gait analysis, we found a relationship between K/L score and 3D kinematic gait for patients. All KOA knees had a significantly reduced range of motion. As the K/L score was increasing, the knee flexion at the heel strike and 50% of the stance phase increased while the peak knee flexion in the swing phase decreased. In addition, the adduction and femoral rotation increased internally at the heel strike, 50% of the stance phase, and maximum angle of the swing phase. Femoral translation increased anteriorly and distally at the heel strike and 50% of the stance phase. The severe group had more medial translation than the asymptomatic groups. Significant alterations of three-dimensional joint kinematics were identified in subjects suffering various severities in Chinese patients. This study provides an important reference for the treatment options, therapy assessment, and rehabilitation of KOA.

## Introduction

KOA is a worldwide degenerative disease characterized by knee pain, loss of articular cartilage, joint stiffness, sclerosis, and osteophytes. KOA can lead to dysfunction and deformity^1^. Among adults aging over 60, 10% of males and 13% of females are affected by symptomatic KOA in the United States^2^. The prevalence of the disease is 7.2% in the middle-aged and elderly Chinese (9.8% and 3.7% for females and males, respectively)^3, 4^. Among the three compartments of the knees, KOA affects the medial compartment most often^5, 6^.

In the past decade, imaging has often been applied to assess KOA clinically, especially radiography^7, 8^. The Kellgren & Lawrence (K/L) system for grading the severity of osteoarthritis was developed based on radiography^7^. At present, K/L scores have been widely applied to the assessment of the severity of KOA in clinics. Computed tomography (CT), magnetic resonance imaging (MRI), and ultrasound (UT) have also been used to evaluate KOA^9–11^. However, these imaging techniques assess KOA under static conditions and, thus, do not evaluate the functional changes of the disease. Hence, gait analysis, a dynamic method, has been used to explore kinetic and kinematic characteristics of KOA for decades^12–16^. For example, the knee adduction moment (KAM) has been a significant parameter correlated with medial KOA^17^. Reduced knee flexion at the heel strike, reduced knee abduction angle at the 50% of the stance phase, and reduced knee flexion range during gait were reported in patients with KOA^18–20^. However, so far, no study has focused on the relationship between six degrees of freedom (6DOF, including flexion/extension, adduction/abduction, internal/external femoral rotation, anteroposterior translation, proximal/distal translation and medial/lateral translation of the knee), and the imaging assessment of the severities in KOA patients. For patients of medial KOA, anatomical structures of the knees, such as osteophytes and deformity, were dependent on the severities of KOA and influenced kinematic alterations of the joints^21, 22^. Hence, this study aims to explore if and how the kinematic alterations of Chinese patients with medial KOA during walking is related to K/L scores quantitatively.

## Results

Demographic and anthropometric characteristics of all the groups are summarized in Table 1. According to K/L criteria^7^, Grade 1, 2 and 3 were classified as early, moderate and severe OA, respectively.

**Table 1:** Mean (±SD) of demographic and anthropometric characteristics of the participants.

Group	Early OA	Moderate	Severe OA	Asymptomatic
Number (male: female)	N = 26 (12:14)	N = 33 (13:20)	N = 38 (10:28)	N = 39 (21:18)
Age(year)	56.3 (10.2)	58.8 (11.6)	63.5 (8.0)	43.3 (5.9)
Weight(kg)	64.2 (10.4)	63.0 (11.3)	58.5 (9.3)	67.6 (10.3)
Height(cm)	163.8 (8.0)	164.4 (10.0)	157.5 (7.9)	165.8 (9.6)
BMI(kg/m^2^)	24.0 (2.4)	23.2 (3.0)	23.6 (3.3)	23.1 (3.5)

**Figure 1:**
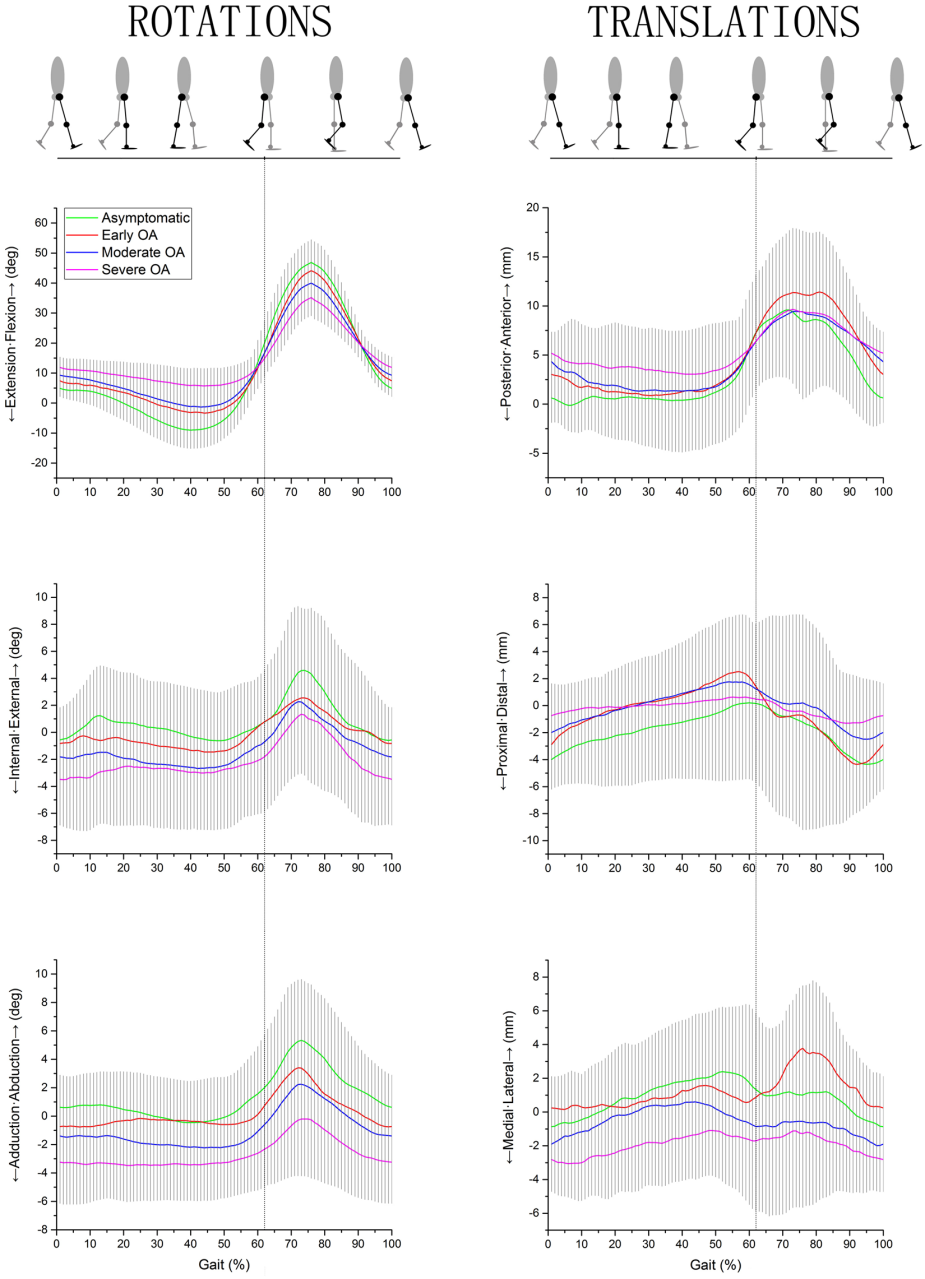
Tibiofemoral kinematics of the subjects in different groups during gait analysis. The thick solid lines represent the participants’ average kinematics of the femur relative to the tibia and the gray shadow area represents the standard deviation of the participants. The green, red, blue and pink lines represent the average motion of the asymptomatic group, early OA group, moderate OA group and severe OA group, respectively. The gait begins with the heel strike and ends with the next heel strike. The gait cycle is divided into a stance phase and a swing phase by toe off (the vertical dashed line, about 62% gait).

**Figure 2:**
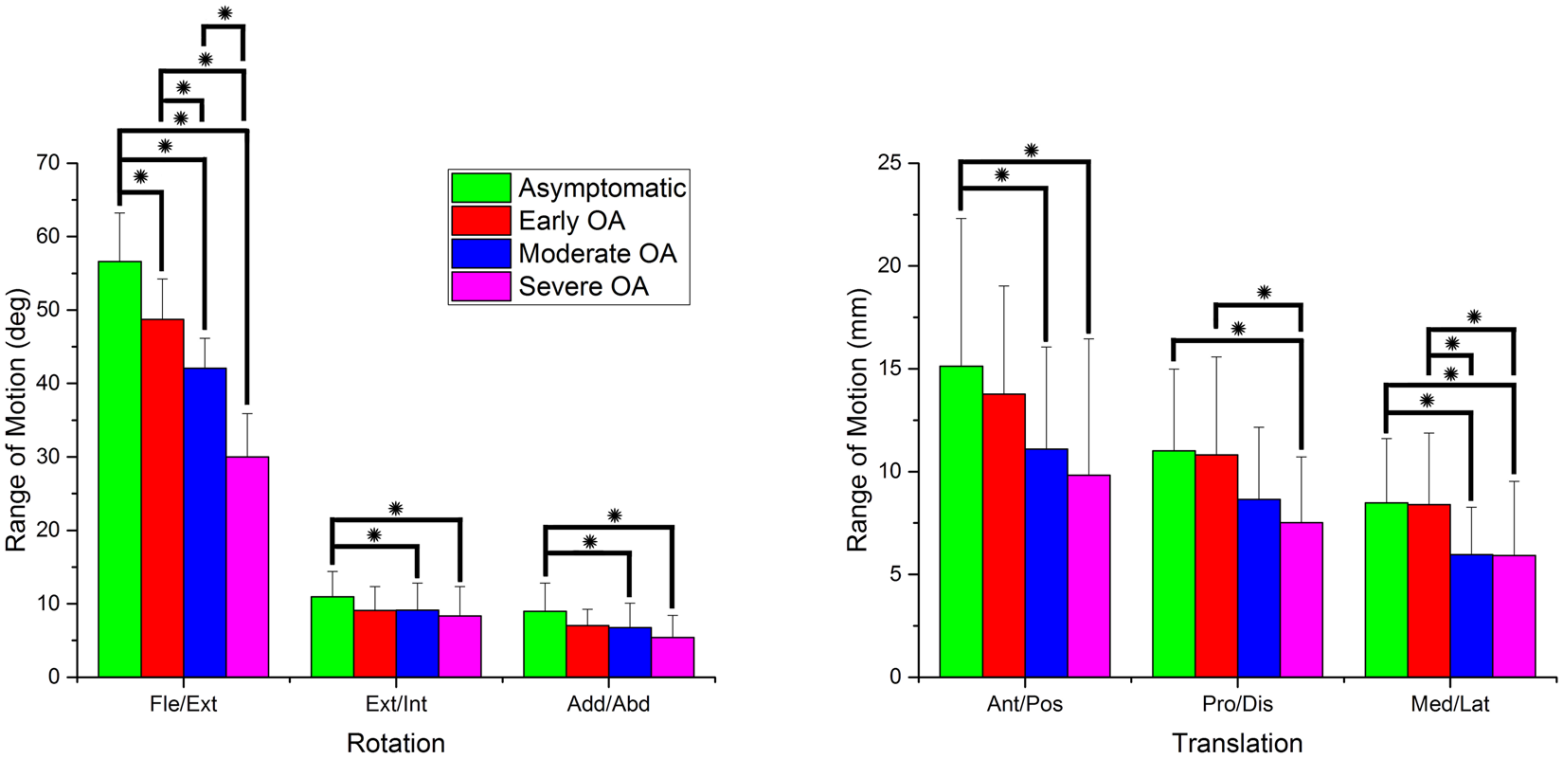
The range of motion of different groups. The green, red, blue and pink bars represent the average motion of the asymptomatic group, early OA group, moderate OA group and severe OA group, respectively. The symbol “*” represents a significant difference between groups (p < 0.05). The figure briefly shows a reducing range of motion in an order of the asymptomatic group, early OA group, moderate OA group and severe OA group.

The mean ± standard deviation (SD) of 6DOF of the subjects during treadmill gait (femur relative to tibia) is shown in Fig. 1. The changes in the range of motion during gait are shown in Fig. 2. The mean ± SD and the comparisons among the asymptomatic participants and OA patients are shown in Tables 2 and 3, respectively.

**Table 2:** Mean ± SD of angular kinematic parameters between knee OA severities (in degrees).

Groups	Asymptomatic (deg) (Group 1)	Early OA (deg) (Group 2)	Moderate OA (deg) (Group 3)	Severe OA (deg) (Group 4)	F	Multiple comparisons p-VALUE (Bonferroni)					
						Group 1 vs 2	Group 1 vs 3	Group 3 vs 4	Group 2 vs 3	Group 2 vs 4	Group 3 vs 4
Flexion/Extension at Heel Strike	4.9 ± 2.7	7.4 ± 2.3	9.3 ± 3.2	11.9 ± 3.5	<0.001	0.007	<0.001	<0.001	0.124	<0.001	0.003
Flexion/Extension at 50% of the Stance Phase	−4.7 ± 6.0	−1.0 ± 7.1	1.1 ± 5.4	7.2 ± 5.5	<0.001	0.087	<0.001	<0.001	1	<0.001	0.001
Maximum Flexion in Swing Phase	46.9 ± 7.6	44.1 ± 8.3	40.0 ± 7.2	35.1 ± 6.0	<0.001	0.813	0.001	<0.001	0.188	<0.001	0.034
ROM of Knee Flexion	56.6 ± 6.6	48.7 ± 5.5	42.1 ± 4.1	30.0 ± 5.9	<0.001	<0.001	<0.001	<0.001	<0.001	<0.001	<0.001
Abduction/Adduction at Heel Strike	0.6 ± 2.3	−0.7 ± 2.2	−1.4 ± 2.3	−3.2 ± 2.9	<0.001	0.192	0.004	<0.001	1	<0.001	0.014
Abduction/Adduction at 50% of the Stance Phase	−0.1 ± 2.8	−0.3 ± 2.6	−2.0 ± 2.8	−3.4 ± 2.5	<0.001	1	0.017	<0.001	0.084	<0.001	0.165
Maximum Abduction in Swing Phase	5.3 ± 4.3	3.4 ± 3.1	2.2 ± 3.7	−2.3 ± 3.9	<0.001	0.355	0.007	<0.001	1	<0.001	0.041
ROM of Knee Abduction/Adduction	9.0 ± 3.9	7.1 ± 2.2	6.7 ± 3.3	5.4 ± 3.0	<0.001	0.126	0.029	<0.001	1	0.276	0.456
Rotation at Heel Strike	−0.6 ± 2.4	−0.8 ± 2.5	−1.8 ± 3.0	−3.5 ± 3.4	<0.001	1	0.410	<0.001	1	0.003	0.103
Rotation at 50% of the Stance Phase	0.3 ± 3.5	−1.0 ± 3.8	−2.4 ± 4.3	−2.7 ± 4.4	0.007	1	0.036	0.009	1	0.683	1
Maximum Rotation in Swing Phase	4.6 ± 4.6	2.5 ± 4.1	2.6 ± 4.9	1.3 ± 4.4	0.019	0.487	0.014	0.014	1	1	1
ROM of Knee Rotation	11.0 ± 3.4	9.1 ± 3.2	9.1 ± 3.7	8.3 ± 4.0	0.016	0.270	0.012	0.012	1	1	1

In this table, the key events of the gait at the heel strike and 50% of the stance phase, the maximum angle at the swing phase, and the range of motion were compared. The multiple statistical differences between groups were performed by Bonferroni correction.

**Table 3:** Mean ± SD of translation of femur relative to tibia between knee OA severities during gait (in mm).

Groups	Asymptomatic (mm) (Group 1)	Early OA (mm) (Group 2)	Moderate OA (mm) (Group 3)	Severe OA (mm) (Group 4)	F	Multiple comparisons p-VALUE (Bonferroni)					
						Group 1 vs. 2	Group 1 vs. 3	Group 1 vs. 4	Group 2 vs. 3	Group 2 vs. 4	Group 3 vs. 4
Anterior/Posterior Translation at Heel Strike	0.6 ± 2.5	3.0 ± 2.2	4.3 ± 2.1	5.2 ± 2.2	<0.001	<0.001	<0.001	<0.001	0.189	0.002	0.673
Anterior/Posterior Translation at 50% of the Stance Phase	0.6 ± 4.9	0.9 ± 3.9	1.4 ± 4.0	3.5 ± 4.3	0.020	1	1	0.022	1	0.119	0.287
Maximum Anterior/Posterior Translation in Swing Phase	8.4 ± 7.9	11.2 ± 6.5	9.4 ± 5.6	9.4 ± 5.9	0.450	0.636	1	1	1	1	1
ROM of Anterior/Posterior Translation	15.1 ± 7.1	13.8 ± 5.3	11.1 ± 5.0	9.8 ± 6.6	0.001	1	0.040	0.001	0.613	0.080	1
Distal/Proximal Translation at Heel Strike	−4.0 ± 2.2	−2.9 ± 1.8	−2.0 ± 2.0	−0.7 ± 2.4	<0.001	0.263	0.001	<0.001	0.697	0.001	0.103
Distal/Proximal Translation at 50% of the Stance Phase	−1.6 ± 4.0	0.3 ± 3.4	0.3 ± 2.9	0 ± 2.7	0.044	0.168	0.091	0.195	1	1	1
Maximum Distal/Proximal Translation During Gait(55%gait)	0 ± 5.5	2.4 ± 4.1	1.7 ± 3.7	0.6 ± 3.9	0.118	0.181	0.560	1	0.671	1	1
ROM of Distal/Proximal Translation	11.0 ± 4.0	10.8 ± 4.8	8.6 ± 3.5	7.5 ± 3.2	<0.001	1	0.059	0.001	0.199	0.006	1
Medial/Lateral Translation at Heel Strike	−0.9 ± 2.0	0.2 ± 1.9	−1.9 ± 1.9	−2.8 ± 1.9	<0.001	0.151	0.154	<0.001	<0.001	<0.001	0.287
Medial/Lateral Translation at 50% of the Stance Phase	1.3 ± 3.2	0.8 ± 3.0	0.4 ± 2.7	−1.8 ± 2.6	<0.001	1	0.950	<0.001	1	0.003	0.010
Maximum Medial/Lateral Translation in Swing Phase	1.0 ± 4.1	3.8 ± 3.7	−0.5 ± 3.1	−1.3 ± 4.4	<0.001	0.034	0.566	0.068	<0.001	<0.001	1
ROM of Medial/Lateral Translation	8.5 ± 3.1	8.4 ± 3.5	6.0 ± 2.3	5.9 ± 3.6	<0.001	1	0.006	0.003	0.024	0.015	1

In this table, the key events of the gait at the heel strike and 50% of the stance phase, the maximum translation at the swing phase as well as the range of motion were compared. The multiple statistical differences between different groups were performed by Bonferroni correction.

### The range of motion

The range of motion was significantly reduced in the following order for the asymptomatic subjects, early OA group, moderate OA group, and severe OA group (Fig. 1). The range of knee flexion decreased from 56.6° to 30.0° during the progress of KOA (p < 0.001). In varus/valgus, the range of motion decreased from 9.0° to 5.4° (p < 0.001). The range of rotational motion decreased from 11.0° to 8.3° (p < 0.05). The range of anteroposterior translation reduced from 15.1 mm to 9.8 mm (p < 0.001). The range of proximal-distal translation decreased from 11.0 mm to 7.5 mm (p < 0.001). The range of medial-lateral translation decreased from 8.5 mm to 5.9 mm (p < 0.001).

### Tibiofemoral Kinematics

The peak knee flexion of the OA groups at the swing phase was smaller than that of the asymptomatic group (p < 0.001) (Table 2). However, the knee flexion of the OA groups at the heel strike (p < 0.001) and at the 50% of the stance phase (p < 0.001) was larger than that of the asymptomatic group. The adduction of the moderate (p < 0.001) and severe (p < 0.001) groups was larger than that of the asymptomatic group at the heel strike, 50% of the stance phase and maximum abduction of the swing phase. Internal femoral rotation of the severe OA group was larger than that of the asymptomatic group at the heel trike (p < 0.001), 50% of the stance phase (p < 0.01) and maximum rotation of the swing phase (p < 0.05).

The femoral anterior translation of the OA group was larger than that of the asymptomatic group at the heel strike (p < 0.001) (Table 2). The femoral anterior translation of the severe OA group was larger than that of the asymptomatic group at the 50% of stance phase (p < 0.05). Distal femoral translation of the moderate (p < 0.001) and severe (p < 0.001) OA group increased at the heel strike. In addition, the medial femoral translation of the severe OA group was more than that of the asymptomatic group at both the heel strike and 50% of the stance phase (p < 0.001).

## Discussion

Static imaging, especially the radiography, has been often clinically accepted to assess the severity of medial KOA. However, there has been no study focusing on 3D kinematic alterations for patients with the different severities of medial KOA.

There have been studies reporting the kinematics of patients with knee OA during gait^20, 23–27^. In addition, some studies have investigated 6DOF kinematics of knee during gait in other diseases^28, 29^. But only up to 4DOF (flexion/extension, adduction/abduction, internal/external femoral rotation and anterior/posterior translation) kinematics of the knees were studied for KOA^30^. Moreover, very few studies dealt with the kinematic characteristics of Asian subjects with KOA during gait and even in such studies only 3DOF (flexion/extension, adduction/abduction, internal/external femoral rotation) were assessed^20^.

Herein, we made an attempt to explore the kinematic alteration (6DOF) of the different severities of medial KOA based on prior imaging assessments. Our results show that the kinematic alterations of patients with medial KOA was strongly correlated with the K/L scores with reduced range of motion and alternations of quantitative kinematic characteristics.

Regarding flexion/extension, our results showed that the range of knee flexion and the peak knee flexion at the swing phase decreased while the knee flexion at both the heel strike and the 50% of the stance phase increased during the progress of knee OA (Table 2). As mentioned before, the patients had pain in their knees. In order to relieve the pain in their knees, the patients would limit their range of motion. Additionally, the patients with KOA were commonly accompanied by the flexion deformity^1^. Hence, these kinematic trends probably resulted from the knee pain and flexion deformity.

Regarding adduction/abduction, our study showed that patients with OA had a smaller range of motion, an increased adduction at the heel strike and 50% of the stance phase, and a decreased maximum abduction at the swing phase (Table 2). Our study exhibited that the angle of adduction/abduction at the heel strike and 50% of the stance phase as well as at the maximum angle of the swing phase decreased in the order of early, moderate, and severe KOA. Additionally, patients with KOA usually have a varus deformity^1^. Hence, our results could indicate that the angle of adduction/abduction is a parameter for the prediction of the severity of KOA. In addition, the reduced range of adduction/abduction could result from the narrow space in the joint, which can limit the motion of the knee.

In axial tibial rotation, our results demonstrated that the patients with medial KOA exhibited an increase in the internal femoral rotation at the heel trike, 50% of the stance phase, and the maximum rotation of the swing phase when KOA became more severe (Table 2). In addition, the range of rotational motion decreased with the severity of KOA. Increased internal rotation of the femur may arise from the rotational deformity of the knee, which would change the location of the femur relative to the tibia.

Regarding anterior/posterior translation, our results showed that the subjects had increased femoral anterior translation at the heel strike and mid-stance phase as the severity of KOA increased (Table 3). We also found that the range of anterior-posterior motion was smaller in patients with KOA. It has been found that the patients with medial KOA exhibited femoral anterior translation and limitation of anterior-posterior motion using magnetic resonance imaging (MRI) by Scarvell *et al*.^31^. It could be related to the deficiency of the anterior cruciate ligament (ACL) since deficiency of ACL can lead to anterior femoral translation in the knees. Alternatively, it could be related to the severity of osteophytes and narrow space as they can affect and lead to the limitation of motion and deformity in the knees. Further study is needed to compare the kinematics of patients with medial knee OA considering the osteophytes and state of ACL.

Regarding proximal/distal translation, our study showed that the range of proximal-distal translation decreased with the progression of knee OA (Table 3). Nishino *et al*. used radiography to discover that the patients with KOA had a smaller range of proximal-distal translation of the tibia^32^, which is in agreement with our study. This result may be caused by the narrowing compartment of the joints. In addition, the distal femoral translation increased at the heel strike in the order of the severities of KOA, probably due to the decreasing thickness of the cartilage and the conditions of the menisci. The moderate amount of cartilage has an elastic property. As KOA progresses, the thickness of the cartilage of the patients would decrease, eventually leading to the exposure of the joints. Thus, when the patients’ feet “land” on the ground, their knees would lack the elastic property needed to relieve the pressure from the ground as a result of decreasing distal femoral translation at the heel strike.

In our results, the range of medial-lateral translation was smaller due to the narrowing compartment of the joints (Table 3). In addition, the medial femoral translation was increased in severe OA. It may be because the lateral collateral ligament (LCL) becomes flabby due to the medial varus moment during the progression of KOA.

So far, there has been no study on the relationship between the static assessment of radiography and the dynamic gait analysis with 6DOF during walking. Through our study, we found a strong relationship between K/L score and 6DOF gait analysis (Table 3). The K/L score determined from radiography mainly reflects the osteophytes and space of the joints^7^ and thus cannot be used to evaluate the outcomes of conservative treatment. A combination of radiography and gait analysis could provide guidance for the conservative treatment for patients with early and moderate KOA without the need of arthroplasty. The use of gait analysis systems in the laboratory usually requires much labor and time. However, our portable gait system can complete the characterization of the kinematics of one patient within 10 min by two doctors, making it possible for clinical doctors to objectively and quantitatively assess KOA and conveniently make a better decision in a busy situation.

There are several limitations in this study. First, our study only focuses on the relationship between K/L score and the alternations of kinematics. Second, the gait system lacks a force plate or a pressure sensor that can help us obtain more information about changes of force in knees. Third, only 136 subjects were recruited in this study. The sample size is not large enough to represent common kinematic characteristics of medial KOA based on K/L score. Fourth, our methods are limited by radiography that cannot detect the soft tissue in knees (e.g., the ACL and meniscus). Fifth, the symptomatic subjects are generally younger than those of OA groups and, thus, the study cannot exclude the factor of age. Sixth, soft tissue artifact (STA) may be distributed on the lower limbs during treadmill gait. In fact, we reduced the STA by a modified marker cluster wrapping procedure^33^ and validated the average translational accuracy to be 2.3 mm. Seventh, the gait pattern on the treadmill may be different from that over the ground. However, some reports declared that only minimal differences were found between treadmill and overground gait in both kinetic and kinematic parameters^34, 35^.

In summary, we found that there is a relationship between K/L score and 3D kinematic gait of patients with medial KOA. We also determined that a reduced range of motion and a series of kinematic alterations reflect the kinematic alterations of the natural history of KOA. This study provides an important reference relevant to the options of treatment, assessment of therapy and rehabilitation of KOA.

## Methods

### Participants

97 patients diagnosed with medial KOA and 38 asymptomatic participants were recruited. The asymptomatic participants (>35 years) had no history of knee pain, trauma, surgery, or obvious gait abnormalities. All participants had a body mass index of less than 35. Clinical physical exams were taken to examine the asymptomatic subjects to avoid knee diseases.

Patients with medial KOA (>35 years) were diagnosed according to the American College of Rheumatology criteria^36^ by an orthopedic surgeon. All recruits had radiographic evidence and medial knee pain. They were divided into three groups based on K/L score^7^, by which the OA was classified into four grades. Specifically, the categorization was set according to the K/L score, including early OA (K/L Grade 1), moderate OA (K/L Grade 2), and severe OA (K/L Grade 3 and 4). Exclusion criteria included the following cases: traumatic OA, lateral tibiofemoral KOA or any other types of Knee OA; ambiguity of K/L scores; surgery, neuromuscular disease, cardiovascular disease or trauma that can affects gait; body mass index greater than 35; needing stick or assistance to walk or being unable to walk at a speed of less than 2.0 km/h and less than 300 m. This study was approved by the Institutional Review Board of Guangzhou General Hospital of Guangzhou Military Command, and informed consents were obtained from all participants. It was conducted in accordance with the principles outlined in the Declaration of Helsinki.

### Gait Analysis

**Figure 3:**
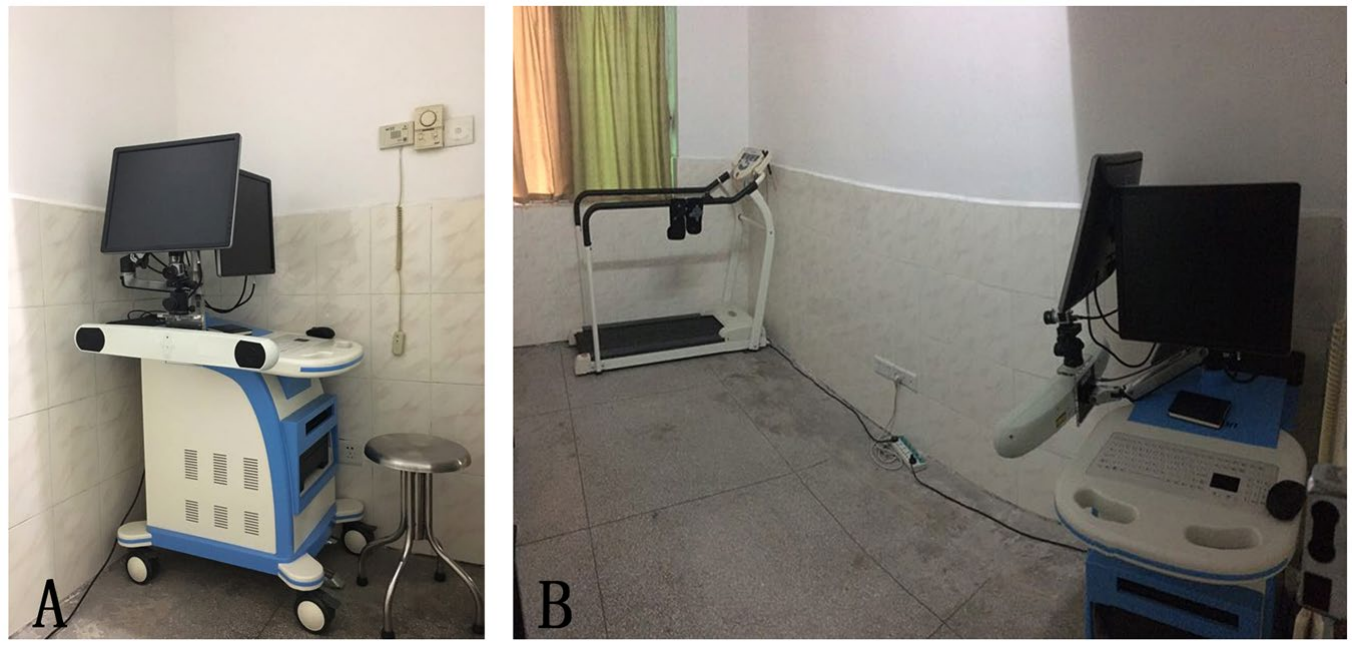
The gait system and the working place. (**A**) The gait analysis machine has about an area of 0.8 m × 0.8 m × 1.5 m. (**B**) The working place is located in the hospital near the doctors’ office for the doctors to test patients.

**Figure 4:**
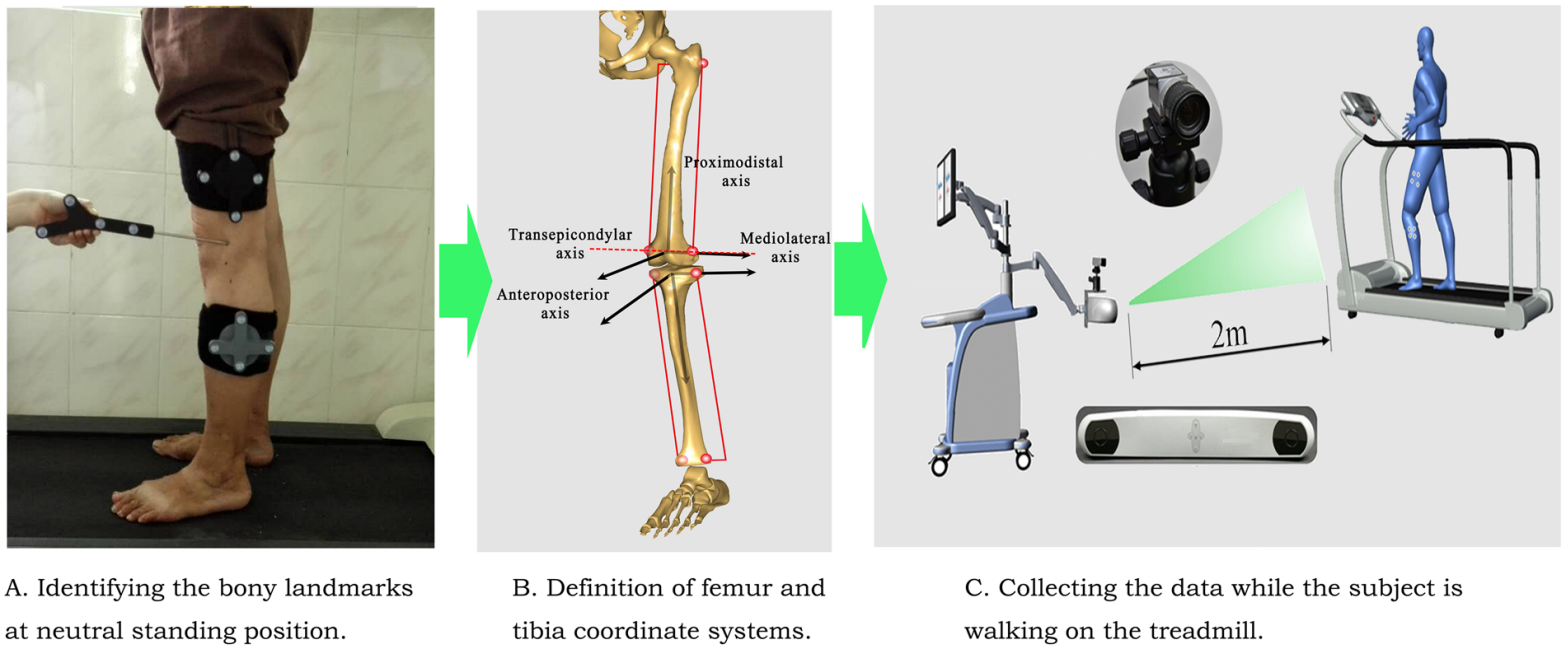
The process of gait analysis. (**A**) The doctor is holding the hand-held digitizing probe to identify the nine bony landmarks (greater trochanter, lateral epicondyle, medial epicondyle, lateral plateau, medial plateau, tibial tuberosity, fibular head, medial malleolus, and lateral malleolus). (**B**) According to the nine bony landmarks, the system sets up the 3D position of tibia and femur. (**C**) The gait system is collecting trajectories of femur relative to tibia while the subjects are walking in the treadmill.

The kinematic data of the participants’ knees was recorded with a marker-based 3D portable gait analysis system (Opti Knee, Innomotion Inc., Shanghai, China)^37^. The system required an area of 2 m × 3 m × 2.5 m and consisted of two markers, two high-speed inferred cameras, a hand-held digitizing probe, a bi-directional treadmill, and a workstation computer (Fig. 3). Two markers were fastened to the middle of thigh and the middle of calf of each participant with bandages with the participant at neutral standing position, respectively. To capture the 3D position and trajectories of the knees, the system had two high-speed infrared cameras at a frequency of 60 Hz. We used a hand-held digitizing probe to identify nine bone landmarks to set the 3D position of the tibia and the femur, and collected trajectories of the femur relative to the tibia (Fig. 4). Finally, we calculated the kinematic data (6DOF) with the workstation computer as the participants walked on the treadmill, including flexion/extension, adduction/abduction, internal/external femoral rotation, anteroposterior translation, proximal/distal translation and medial/lateral translation of the knees.

Before data collection, each participant was trained to adapt to walk on the treadmill. Hence, the participants could walk on the treadmill at 2.0 km/h as if they were walking on the ground. Finally, we recorded the data for 15 s of gait at a speed of 2.0 km/h. The total testing time for each participant was within 10 min.

6DOF were calculated from the trajectories of the femur relative to the tibia. The average from all gait cycles of each participant was calculated and divided into two phases: stance phase (~62% of the gait) and swing phase (~38% of the gait)^38^. We calculated the range of motion of 6DOF and knee kinematics at the time of heel strike, 50% of the stance phase and maximum motion of 6DOF at the swing phase, which reflected the status of the knee kinematics in patients with knee OA^30, 38^. We compared the differences using One-Way Analysis of Variance (ANOVA). The Bonferroni correction was performed between groups when significant differences were detected.
